# Genome variability of domestic tomato varieties: data from AFLP analysis

**DOI:** 10.18699/VJGB-22-80

**Published:** 2022-11

**Authors:** A.V. Kulakova, E.A. Dyachenko, A.V. Shchennikova, O.N. Pyshnaya, E.A. Dzhos

**Affiliations:** Institute of Bioengineering, Research Center of Biotechnology of the Russian Academy of Sciences, Moscow, Russia; Institute of Bioengineering, Research Center of Biotechnology of the Russian Academy of Sciences, Moscow, Russia; Institute of Bioengineering, Research Center of Biotechnology of the Russian Academy of Sciences, Moscow, Russia; Federal Scientific Vegetable Center, VNIISSOK, Moscow region, Russia; Institute of Bioengineering, Research Center of Biotechnology of the Russian Academy of Sciences, Moscow, Russia Federal Scientific Vegetable Center, VNIISSOK, Moscow region, Russia

**Keywords:** Solanum lycopersicum, tomato cultivars, genomic polymorphism, AFLP markers, clustering, Solanum lycopersicum, сорта томата, геномный полиморфизм, AFLP-маркеры, кластеризация

## Abstract

Tomato Solanum lycopersicum L. is one of the main vegetable crops, accessions and cultivars of which are characterized by a low level of genomic polymorphism. Introgressive tomato breeding uses related wild Solanum species to improve cultivars for stress tolerance and fruit quality traits. The aim of this work was to evaluate the genome variability of 59 cultivars and perspective breeding lines of S. lycopersicum and 11 wild tomato species using the AFLP method. According to the AFLP analysis, four combinations of primers E32/M59, E32/M57, E38/M57, and E41/M59, which had the highest PIC (polymorphism information content) values, were selected. In the process of genotyping a collection of 59 cultivars/lines of S. lycopersicum and 11 wild tomato accessions, the selected primers revealed 391 fragments ranging in size from 80 to 450 bp, of which 114 fragments turned out to be polymorphic and 25 were unique. Analysis of the amplif ication spectra placed wild tomato accessions into separate clades. Sister clades included cultivars of FSCV breeding resistant to drought and/or cold and, in part, to late blight, Alternaria, Septoria, tobacco mosaic virus and blossom end rot, as well as tomato accessions not characterized according to these traits, which suggests that they have resistance to stress factors. In accessions of distant clades, there was clustering on the basis of resistance to Verticillium, cladosporiosis, Fusarium, tobacco mosaic virus, gray rot, and blossom end rot. The combination of ac cessions according to their origin from the originating organization was shown. The primer combinations E32/M59, E32/M57, E38/M57 and E41/M59 were shown to be perspective for genotyping tomato cultivars to select donors of resistance to various stress factors. The clade-specif ic fragments identif ied in this work can become the basis for the development of AFLP markers for traits of resistance to stress factors.

## Abstract (ru)

Томат Solanum lycopersicum L. является одной из основных овощных культур, образцы и сорта которой характеризуются низким уровнем геномного полиморфизма. В интрогрессивной селекции томата используют родственные дикорастущие виды Solanum для улучшения сортов по признакам устойчивости к стрессовым факторам и качества плодов. Целью работы была оценка вариабельности генома 59 сортов и перспективных селекционных линий S. lycopersicum и 11 дикорастущих видов томата с помощью метода AFLP. По данным AFLP-анализа было выбрано четыре комбинации праймеров E32/M59, E32/M57, E38/M57 и Е41/М59, которые отличались наиболее высокими показателями PIC (polymorphism information content). В процессе маркирования коллекции из 59 сортов/линий S. lycopersicum и 11 дикорастущих образцов томата отобранными праймерами выявлен 391 фрагмент размером от 80 до 450 п. н., из которых 114 фрагментов оказались полиморфными и 25 – уникальными. Анализ спектров амплификации выделил дикорастущие образцы томата в отдельные клады. Сестринские клады включали сорта селекции Федерального научного центра овощеводства, устойчивые к засухе и/или холоду и, частично, к фитофторозу, альтернариозу, септориозу, вирусу табачной мозаики и вершинной гнили плода, а также не охарактеризованные по данным признакам образцы томата, что позволяет предположить наличие у них устойчивости к стрессовым факторам. У сортовых образцов отдаленных клад присутствует кластеризация по признакам устойчивости к вертициллезу, кладоспориозу, фузариозу, вирусу табачной мозаики, серой гнили и вершинной гнили плода. Показано объединение образцов согласно их происхождению от организации-оригинатора. Продемонстрирована перспективность праймерных комбинаций E32/M59, E32/M57, E38/M57 и Е41/М59 для генотипирования сортов томата с целью отбора доноров устойчивости к различным стрессовым факторам. Выявленные в настоящей работе кладоспецифичные фрагменты могут стать основой для разработки AFLP-маркеров для признаков устойчивости к стрессовым факторам.

## Introduction

The assessment of genetic diversity, considering the pedigrees of crop cultivars and associations with important traits, is one of the foundations of modern breeding. Various methods of molecular genome analysis are used in the selection of parental genotypes, as well as in identifying the level of variability both within a variety and between varieties (Nurmansyah et al., 2020; Sheeja et al., 2021). Both the entire plant genome and its particular regions (gene families, specific loci, individual genes) are subjected to DNA genotyping. Polymorphism data are used, for example, to develop molecular DNA markers linked to important traits. Markers are used to search for donors of the corresponding genotypes, as well as to certify varieties and lines (Semagn et al., 2006; Swiecicka et al., 2009).

One of the commonly used methods for assessing plant genome variability is the AFLP (Amplified Fragment Length Polymorphism), which is based on the assessment of unique and moderately repetitive genome sequences, but does not require the determination of the sequences themselves (Vos et al., 1995; Karp et al., 1997; Despres et al., 2003). The evaluation is based on selective PCR amplification of restriction fragments from a total genomic DNA digest (Vos et al., 1995). The use of AFLP markers is applicable to all species, highly reproducible, and highly efficient in determining genetic distances and phylogenetic relationships in taxonomy (Kardolus et al., 1998; Mba, Tohme, 2005; Arif et al., 2010). The method has been successfully applied to study wild and endangered plant species (Zawko et al., 2001; Ronikier, 2002; van Ee et al., 2006; Manoko et al., 2007; Elameen et al., 2008; Li et al., 2008; Sánchez-Teyer et al., 2009; Tatikonda et al., 2009). In addition, AFLP is popular in modern plant breeding and is used to determine pedigrees, variability, homogeneity, and the degree of introgression and hybridity of varieties, as well as to search for molecular markers associated with economically valuable traits (Mba, Tohme, 2005; Swiecicka et al., 2009; Arif et al., 2010). Such studies have been carried out, for example, on wheat (Hassan et al., 2018), barley (El-Esawi et al., 2018a), peas (D’iachenko et al., 2014; El-Esawi et al., 2018b), pepper (Kochieva, Ryzhova, 2009) and potato (McGregor et al., 2002; Jacobs et al., 2008; Bamberg, del Rio, 2014; Bryan et al., 2017; Dyachenko et al., 2020).

The AFLP has also been used for genotyping tomato (Solanum lycopersicum L.). Thus, with this method, an intraspecific map of the tomato genome was obtained (Saliba-Colombani et al., 2000), the transcriptional response of tomato to nematode infection was studied (Święcicka et al., 2017), and DNA markers linked to resistance to tomato bacterial wilt (Miao et al., 2009) and cladosporiosis (Thomas et al., 1995) were identified. The use of AFLP for comparing the response of heat-tolerant and heat-sensitive tomato genotypes to moderate heat stress conditions revealed a number of differentially expressed constitutive genes, presumably determining heat tolerance and differences in genotype adaptation to elevated temperatures (Bita et al., 2011).

The phylogenetics and genogeography of crop wild relatives are effective approaches to understanding their evolutionary patterns and unlocking their potential to improve crops. AFLP genotyping against geographic and climatic indicators has contributed to the study of the spatial genetics of wild tomato species S. lycopersicum, S. pimpinellifolium (Nakazato, Housworth, 2011) and S. peruvianum (Nakazato et al., 2012). The S. lycopersicum and S. pimpinellifolium evolutionary patterns, including demographic history, dispersal patterns, interspecific divergence and hybridization, have been shown to be closely related to the complex geographic and ecological conditions in the Andes (Nakazato, Housworth, 2011). An AFLP study of 19 natural populations of S. peruvianum revealed a moderate degree of population differentiation, probably reflecting partial geographic isolation between tomato species (Nakazato et al., 2012).

In addition to solving taxonomic and phylogenetic problems, the AFLP method is used to determine the variability of tomato varieties. Various DNA marking systems showed low efficiency for studying the genetic diversity of tomato cultivars with limited genetic variability. The use of AFLP in combination with SSR markers to characterize 48 closely related Spanish tomato varieties made it possible to obtain a unique fingerprint for each analyzed accession (García-Martínez et al., 2006).

Cultivated varieties and lines of tomato belong to the species S. lycopersicum. Compared to wild related species (section Lycopersicon of the genus Solanum) (Peralta et al., 2008), their genomes are significantly less polymorphic (20 or more times) (The 100 Tomato Genome Sequencing Consortium et al., 2014). Hundreds of genes and loci of quantitative traits linked to resistance, yield, flower and fruit characteristics, and plant architecture have been mapped in the genome of wild species (Foolad, 2007). Due to the relative ease of crossing with S. lycopersicum, wild species are actively used in introgressive tomato breeding to improve economic traits associated with stress resistance, yield and quality (Hajjar, Hodgkin, 2007; Labate, Robertson, 2012). For example, sources of varying degrees of resistance to bacterial wilt are L. pimpinellifolium (= S. pimpinellifolium) PI127805A, L. esculentum var. cerasiforme (= S. lycopersicum var. cerasiforme) CRA66, L. pimpinellifolium PI129080 and L. esculentum AS52 (Chellemi et al., 1994). In cultivars with purple fruits, the trait of anthocyanin biosynthesis in the fruit was obtained by introgression from the genomes of wild species S. chilense and S. cheesmaniae (Povero et al., 2011; Maligeppagol et al., 2013).

Thus, the low level of genomic polymorphism of tomato varieties is combined with introgressive genes/loci associated with economically valuable traits. Therefore, multilocus genome mapping methods can presumably separate cultivars according to useful traits.

Despite the importance of varietal certification and assessment of intervarietal genome variability, there are few studies on marking the genotypes of tomato cultivars in Russia, and these are mainly works on genotyping using already known markers (Shcherban, 2019). For example, a collection of tomato varieties and hybrids from the Michurinsky State Agrarian University was screened using the P7 molecular marker to identify donors of cladosporiosis resistance (Shamshin et al., 2019).

In this study, using the AFLP method, we assessed the genomic variability of tomato S. lycopersicum cultivars and lines of domestic and foreign breeding from the collection of the Federal Scientific Vegetable Center (FSVC) in comparison with wild accessions of tomato species.

## Materials and methods

For the study, 59 tomato S. lycopersicum cultivars and perspective breeding lines of domestic and foreign breeding from the FSVC collection were selected (Table 1). 11 wild tomato species were used as an outgroup (see Table 1). 34 varieties of the sample (~58 %) are included in the State Register of Breeding Achievements Approved for Use of the Russian Federation for 2022 (https://reestr.gossortrf.ru/). Seeds of accessions were germinated under standard greenhouse conditions (23 °С/25 °С, 16 h/8 h – day/night). Genomic DNA was isolated from freshly harvested 5–6 day old seedlings using the CTAB method (Puchooa, 2004).

**Table 1.**
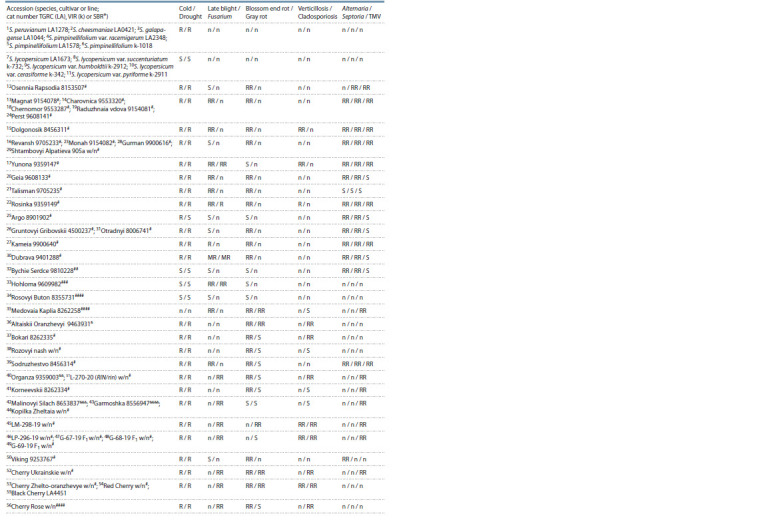
Tomato accessions used for AFLP analysis and their resistance to various stresses

**Table 1end.**
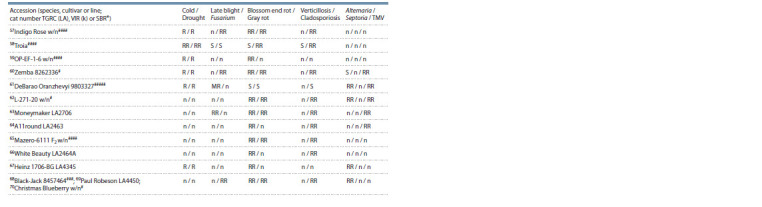
Tomato accessions used for AFLP analysis and their resistance to various stresses Abbreviations: w/n – without number; n – no data; R – resistant (<0.5 score), RR – relatively resistant (0.5–1.0), MR – moderately resistant (1.1–2.0); S – sensitive (>2.0). Late blight (Phytophthora infestans de Bary A); Fusarium (Fusarium oxysporum (Schlecht.) f. sp. lycopersici (Sacc.)); Verticillosis (Verticillium alboatrum and V. dahliaе); Cladosporiosis (Cladosporium fulvum Cooke); Gray rot (Botrytis cinerea Pers); Alternaria (Alternaria solani Sorauer); Septoria (Septoria lycopersici Speg); TMV – Tobacco mosaic virus. * According to SBR (State Register of Breeding Achievements; http://reestr.gossortrf.ru/), TGRC – Tomato Genetic Resource Center (https://tgrc.ucdavis.edu/) or VIR (The N.I. Vavilov All-Russian Institute of Plant Genetic Resources). 1–70 Numbering of accessions (used in Fig. 1–3). # FSVC; ## LLC ‘Agrofirm Poisk’; ### LLC ‘Research Institute of Vegetable Breeding’, LLC ‘Agrofirma GAVRISH’; #### LLC ‘Breeding company GAVRISH’; ##### LLC ‘Breeding and seed-growing company ‘Gisok’; & LLC Agrofirma ‘Demetra-Sibir’; && MONSANTO HOLLAND B. V.; &&& LLC ‘Agrofirma Aelita’; &&&& LLC ‘Premium seeds’.

Data on drought and cold resistance, resistance and susceptibility to diseases (late blight, Fusarium, Verticillium, cladosporiosis, alternariosis, Septoria, tobacco mosaic virus, gray rot, blossom end rot) were partially taken from the State Register of Breeding Achievements (http://reestr.gossortrf. ru/), as well as kindly provided by the originators of the varieties and Ph.D. I.A. Engalycheva.

AFLP analysis was carried out according to the standard protocol: hydrolysis of 350 ng of genomic DNA of each accession with restriction enzymes EcoRI and MseI followed by ligation with EcoRI and MseI adapters (Vos et al., 1995). Selective amplification was performed in two stages: (1) preamplification (denaturation at 94 °C for 30 s, primer annealing at 56 °C for 30 s, synthesis at 72 °C for 1 min, 24 cycles) with adapter primers EcoRI+1 and MseI+1 (Vos et al., 1995) with one selective nucleotide (A) at the 3′ end; (2) amplification with primers EcoRI+3 and MseI+3 with three selective nucleotides at the 3′ end. The results were visualized in a denaturing 6 % polyacrylamide gel using a LI-COR 4300 gel analyzer (LI-COR operator manual; LI-COR, USA). The experiment was carried out in one repeat for each combination of primers. The polymorphic information content (PIC) index for each primer combination was calculated according to Botstein et al. (1980) and Krishnamurthy et al. (2015).

Molecular panels of AFLP fragments were documented in the form of binary matrices (Excel program). Based on the constructed spectra and matrices, variety-specific DNA markers were identified, coefficients of pairwise genetic similarity/ difference between accessions (GS) and genetic distances (GD = 1 – GS) were calculated, cluster analysis was performed (Neighbor Joining method; method of principal coordinates, PCA) and groups of genetically similar accessions were determined (PAST software package) (Hammer et al., 2001). Analysis of the genomic structure of the population of the studied accessions was carried out using the Structure v.2.3.4, which makes it possible to identify common genetic blocks and their ratio in each accession (Pritchard et al., 2000; Hubisz et al., 2009).

## Results

Since up to 80 % of the standard AFLP spectrum can serve as markers for the detection of genetic polymorphisms, and the effectiveness of AFLP depends on primer combinations (Vos et al., 1995), primer/enzyme combinations were selected and tested for multilocus AFLP analysis of tomato accessions. On a sample of five tomato accessions, seven combinations of primers EcoRI+3/MseI+3 were tested, differing in the composition of selective nucleotides at the 3′ end: E32/M59 (E-AAC/M-CTA); E32/M57 (E-AAC/M-CGG); E38/M57 (E-ACT/M-CGG); E41/M59 (E-AGG/M-CTA); E32/M61 (E-AAC/M-CTG); E38/M47 (E-ACT/M-CAA); E38/M59 (E-ACT/M-CTA). It was shown that the use of combinations of E32/M59, E32/M57, E38/M57 and E41/M59 gives a polymorphic, well-differentiated spectrum with an optimal number of fragments.

Four selected primer combinations were used to label 59 S. lycopersicum cultivars/lines and 11 wild tomato accessions. As a result, 391 fragments 80–450 bp in size were detected, of which 114 (29.2 %) fragments turned out to be polymorphic (Table 2). The primer combination E41/M59 was the most effective: 47 out of 67 obtained fragments were variable. At the same time, the E32/M59 combination corresponded to the largest number of fragments unique for individual accessions (11 out of 25 found) (see Table 2). In case of the combinations E32/M61, E38/M47, and E38/M59 (the number of obtained fragments was 31, 24, and 41, respectively) no polymorphic and unique fragments were identified. The PIC value ranged from 0.367 (E32/M57) to 0.658 (E41/ M59) (see Table 2) with a mean value of 0.504, indicating that a large number of polymorphisms can be detected using the E41/M59 primer pair.

**Table 2.**
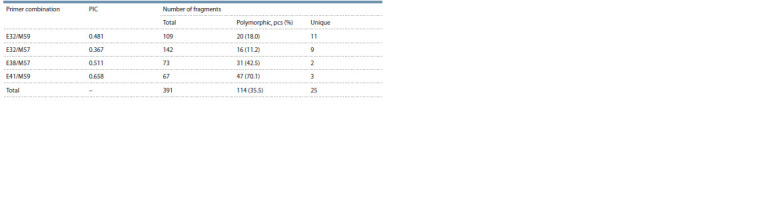
Results of AFLP analysis of tomato species, cultivars, hybrids and lines

**Fig. 1.**
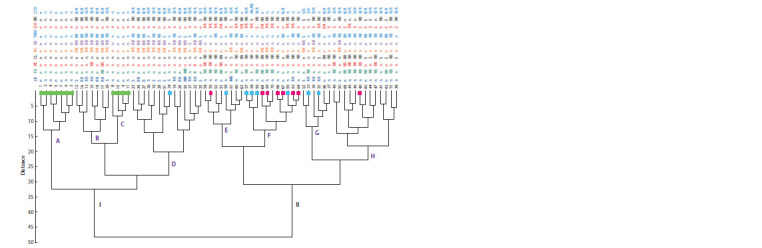
Dendrogram based on AFLP data for cultivated and wild tomato accessions According to Table 1, the accessions are numbered (1–70), and resistance to late blight (LB), Fusarium (FU), Verticillium (VE), cladosporiosis (CL), alternariosis (AL), Septoria (SE), tobacco mosaic virus (TMV), gray rot (GR), blossom end rot (BR), cold (C) and drought (D) is indicated. The degree of resistance of the accessions is given according to Table 1: n – no data, S – susceptible, R – resistant, RR – relatively resistant, MR – moderately resistant. Boxes mark accessions: wild (green), foreign breeding (pink), breeding of LLC ‘Breeding company GAVRISH’ (blue); the rest are breeding of the FSVC.

Based on the results of the AFLP analysis, a dendrogram that clearly divided the tomato accessions into clusters I and II was constructed (Fig. 1).

Wild tomato accessions were grouped into two clades of cluster I: accessions 1 to 7 (including representatives of wild tomato species and a wild accession of S. lycopersicum) were separated into clade A; accessions 8–11, including wild accessions of cultivated species (S. lycopersicum var. succenturiatum, var. humboldtii, var. cerasiforme and var. pyriforme) fell into clade C. Clade C was sister to clade B, consisting of seven S. lycopersicum cultivars (accessions 12–15, 17, 18, and 29; see Table 1, Fig. 1). Clade D (intermediate position between A and B+C) combined 14 tomato varieties/lines. The two clades of cluster II, in turn, were divided into two subclades each (see Fig. 1).

**Fig. 2.**
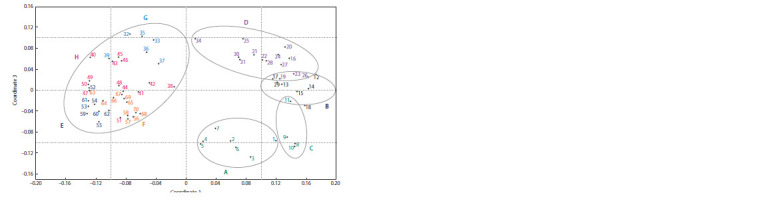
PCA plot of AFLP data for 70 cultivated and wild tomato accessions The numbers correspond to the numbering of accessions in Table 1. The distribution of accessions by clades is shown in accordance with the dendrogram in Fig. 1: clades A and C are highlighted in green, B in black, D in lilac, E in dark blue, F in orange, G in blue, H in pink.

On the graph constructed by the method of principal components, the analyzed cultivars formed three diffuse pools of genotypes, where, as in the dendrogram, a group of wild accessions stood out, and tomato varieties/lines were clustered in a similar way (Fig. 2). There was a clear division between clusters I and II (according to the dendrogram). Wild accession 11 (S. lycopersicum var. pyriforme) was the closest to subclade B varieties/lines.

It was interesting to analyze the possible relationship between the clustering of cultivars and accessions obtained from AFLP data and resistance to various biotic and abiotic stresses.

Varieties/lines of tomato included in cluster I (clades B, D) are the result of breeding by the FSVC (except accession 34). All of them are resistant to cold and/or drought, while accession 34 is susceptible. A similar situation is observed in the case of resistance to blossom end rot, Septoria and Alternaria. All clade B accessions are resistant to tobacco mosaic virus, as are half of clade D accessions (the other half are susceptible). Six accessions of clade D and five accessions of clade B are resistant to late blight; the remaining accessions of these clades are susceptible to this disease

Accessions of subclades E and H, with the exception of one uncharacterized accession (62), are characterized by resistance to cold and drought; in subclades F and G, four and three accessions are resistant, respectively. Subclades E and F are distinguished by resistance to blossom end rot, gray mold and cladosporiosis (except for single susceptible or uncharacterized varieties). About half of subclade E accessions are resistant to Verticillium and Fusarium. Most of subclade H accessions, as well as two groups of the subclade F, are resistant to Fusarium. Subclade G accessions have resistance to late blight (see Fig. 1). Almost all subclade H accessions originated from the FSVC. Accessions of foreign breeding (except for 55 and 40) stand out in subclade F, clustering together with accessions of breeding of LLC ‘Breeding company GAVRISH’.

The study also included an analysis of the population structure of 70 tomato accessions, which revealed common genetic blocks and their ratio in each accession. This distributed the analyzed accessions into clusters. In total, 16 options for the number of subgroups (k) from 3 to 18 were analyzed. The best result (LnLike = –12363.6) was obtained for k = 3.

**Fig. 3.**
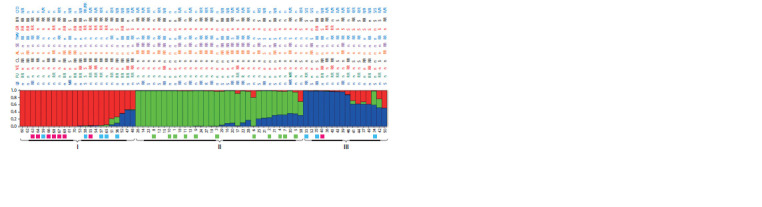
Genomic structure of 59 cultivated and 11 wild tomato accessions according to AFLP analysis (k = 3). According to Table 1, the accessions are numbered (1–70), and the resistance to late blight (LB), Fusarium (FU), Verticillium (VE), cladosporiosis (CL), alternariosis (AL), Septoria (SE), tobacco mosaic virus (TMV), gray rot (GR), blossom end rot (BR), cold (C) and drought (D) is indicated. The degree of resistance of the accessions is given according to Table 1: n – no data, S – susceptible, R – resistant, RR – relatively resistant, MR – moderately resistant. Boxes mark accessions: wild (green), foreign breeding (pink), breeding of LLC ‘Breeding company GAVRISH’ (blue); the rest are breeding of the FSVC.

On the graph, the genomic structure of the studied 70 tomato accessions is presented in the form of various ratios of three blocks (Fig. 3). All accessions of wild species, including accessions of S. lycopersicum, fell into cluster II. An analysis of the correlations between the distribution of accessions by clusters and the traits under consideration (see Table 1) showed a tendency to combine accessions in terms of resistance to gray rot, blossom end rot, Fusarium, cladosporiosis, and Septoria (cluster I). Cold and drought resistant accessions are presented in large numbers in all three clusters. Resistance to Alternaria, Septoria, and TMV proved to be the most typical for cluster II (see Fig. 3). Also, half of the varieties in cluster II are resistant to blossom end rot, and a third of the accessions are resistant to late blight. Cluster III accessions were characterized by different variants of resistance; we can assume clustering on the basis of resistance to TMV (11 out of 16 accessions), as well as susceptibility to gray rot. Except for accession 40 (cluster III), all tomato accessions of foreign breeding were identified in cluster I. The accessions of the LLC ‘Breeding company GAVRISH’ were distributed similarly (four accessions – cluster I, three accessions – cluster III) (see Fig. 3).

## Discussion

In this study, using the AFLP method, we analyzed 11 wild and 59 cultivated (S. lycopersicum) tomato accessions, mainly of domestic breeding (see Table 1). It should be noted that data on resistance to various diseases (Gossortreestr, originators) are unknown for some analyzed cultivated and wild accessions studied. The species S. lycopersicum (wild accessions 7–11 in Table 1) comes from the humid tropics of South America and is a classic example of a cold-sensitive crop (Rick, 1976). The remaining wild species used (accessions 1–6 in Table 1) grow in different climatic zones of South America, from the tropics of the Amazon basin to deserts along the coast and the cold high mountains of the Andes (Nakazato et al., 2010). This suggests that accessions 1–6 are resistant to cold and drought, and accessions 7–11 are sensitive to these stresses.

Each of the 70 accessions was characterized by a specific range of fragments obtained using a combination of four primer pairs (see Table 2). The efficiency obtained (391 fragments, including 114 polymorphic fragments) was comparable with the results of other studies. For example, an AFLP analysis of 21 tomato varieties with four primer combinations revealed 298 fragments, including 159 polymorphs (Suliman- Pollatschek et al., 2002). The percentage of polymorphic fragments obtained by us (29.16 %) also fit into the known data on different crops – in a number of studies it varies from 17.4 to 78.3 % (Kim et al., 1998; Vetelainen et al., 2005).

Analysis of the obtained AFLP data using various bioinformatic methods distributed the studied tomato accessions in a similar way (see Fig. 1–3). Wild tomato accessions isolated themselves into a separate group (see Fig. 2, 3) or divided into clades within cluster I (see Fig. 1). In the dendrogram, accessions 1–6 (tomato species except S. lycopersicum) constituted a separate clade A, and 8–11 (various wild S. lycopersicum accessions) constituted clade C (see Fig. 1). At the same time, accession 7 (S. lycopersicum LA1673) did not combine with 8–11, but entered the subclade with red-fruited accessions 3–6 (S. pimpinellifolium, S. galapagense), which may indicate a probable interspecific introgression. Sister clades B and D consisted of S. lycopersicum cultivars, for which resistance to drought and/or cold was shown (see Fig. 1). This, on the one hand, confirms our assumptions about the possible resistance of wild accessions 1–6 taken for analysis to drought/cold, and also suggests this trait in accessions 7–11. Cold/drought resistance in more than half of the samples of clusters I and II (see Fig. 1) allows us to assume the presence of such resistance in varieties for which there are no data. In addition, the results may indicate the presence of traits of resistance to abiotic stresses introgressed from wild tomato species in the genome of varieties of both clusters.

A fairly clear grouping of accessions by origin shows the effectiveness of the analysis and, at the same time, helps to trace possible links in the pedigree of varieties both from one originator and between breeding centers

## Conclusion

Thus, using AFLP genotyping of selectively neutral regions of the genome of S. lycopersicum cultivars/lines and wild tomato species, clustering of accessions was shown according to resistance to biotic and abiotic stress factors, as well as according to origin from different breeding centers. The prospects of AFLP with the set of primer combinations chosen in this study for genotyping tomato varieties in order to select cultivars with resistance to various stresses were demonstrated. The obtained clade-specific fragments can become the basis for the development of specific molecular markers associated with economically important traits. Sequencing polymorphic AFLP fragments that underlie differences between accession clusters, mapping them on the genome, and assessing the variability of such regions among the analyzed varieties may be promising for obtaining STS markers.

## Conflict of interest

The authors declare no conflict of interest.
